# LncRNA CASC2 is up-regulated in osteoarthritis and participates in the regulation of IL-17 expression and chondrocyte proliferation and apoptosis

**DOI:** 10.1042/BSR20182454

**Published:** 2019-05-14

**Authors:** Tao Huang, Jian Wang, Yuan Zhou, Yi Zhao, Donghua Hang, Yun Cao

**Affiliations:** 1 Department of Orthopaedics, Baoshan Branch of Shanghai General Hospital, Shanghai Jiao Tong University School of Medicine, Shanghai 200940, P.R. China; 2 Department of Orthopaedics, Shanghai General Hospital, Shanghai Jiao Tong University School of Medicine, Shanghai 200080, P.R. China

**Keywords:** apoptosis, chondrocytes, lncRNA CASC2, IL-17, osteoarthritis, proliferation

## Abstract

Long non-coding RNAs (lncRNA) CASC2 is a key player in cancer biology. Our new findings showed that both lncRNA CASC2 and IL-17 were up-regulated in plasma of osteoarthritis patients. Plasma levels of lncRNA CASC2 and IL-17 were significantly and positive correlated only in osteoarthritis patients. Overexpression of lncRNA CASC2 led to up-regulated expression of IL-17 in cells of human chondrocyte cell line CHON-001 (ATCC^®^ CRL-2846™). In addition, overexpression of lncRNA CASC2 inhibited the proliferation, and promoted the apoptosis of chondrocyte. Therefore, lncRNA CASC2 is up-regulated in osteoarthritis and participates in the regulation of IL-17 expression and chondrocyte proliferation and apoptosis.

## Introduction

Osteoarthritis is the most frequently diagnosed chronic joint disease that involves both inflammatory processes and other pathological changes [1], which is different from rheumatoid arthritis, an inflammatory disease [2]. It is generally believed that more than 10% of the population will develop osteoarthritis during their life time [3], leading to a heavy burden on public health. Even worse, prevalence rate of this disease is predicted to be increased in near future due to the growing of old population and increased incidence of overweight or obesity [4]. Osteoarthritis causes pain or even disability in some severe cases. In spite of efforts made on the treatment of this disease [5,6], treatment outcomes are still unsatisfactory, partially due to the unclear pathogenesis [7].

Osteoarthritis is essentially an inflammatory disease [8]. During the development and progression of osteoarthritis, the altered balance between pro-inflammatory and anti-inflammatory cytokines leads to severe inflammatory response in the body of patients [9]. It has been reported that the level of IL-17, which is a pro-inflammatory factor, determines the severity of osteoarthritis [10]. Moreover, in rheumatoid arthritis, IL-17 is an inflammation inducers and contributes to synovium matrix destruction [11].

Long non-coding RNAs, or lncRNAs (>200 nt), are non-protein-coding RNA transcripts with critical roles in human diseases [12,13], including osteoarthritis [14]. LncRNA CASC2 is a well-characterized tumor suppression lncRNA in human cancers [15,16], while its involvement in osteoarthritis is unknown. In the present study we found that lncRNA CASC2 was up-regulated in osteoarthritis and participated in the regulation of IL-17 expression and chondrocyte proliferation and apoptosis.

## Materials and methods

### Specimens

Blood was extracted from 71 osteoarthritis patients and 55 healthy controls at Shanghai General Hospital from July 2016 to July 2018. Patients’ inclusion criteria are as follows: (1) newly diagnosed cases; (2) no therapies initiated. Exclusion criteria are as follows: (1) patients who were treated within 3 months before admission; (2) patients with other diseases, such as chronic inflammatory diseases. Besides that, synovial fluid was extracted from 21 osteoarthritis patients and 15 healthy controls (from the same set of participants aforementioned). The 71 osteoarthritis patients included 45 males and 26 females, and age ranged from 29 to 67 years, with a mean age of 47.6 ± 6.4 years. The 55 healthy controls were selected to match the age and gender distributions of patient group. Control group included 35 males and 20 females, and age ranged from 30 to 66 years, with a mean age of 47.1 ± 6.2 years. The present study passed the review of Shanghai General Hospital. All patients were informed with the details of experimental principle and provided written informed consent about the institutional review board (IRB) protocols.

### RT-qPCR

Plasma, synovial fluid and *in vitro* cultivated cells were mixed with Trizol reagent (Invitrogen, USA) to extract total RNAs. Following reverse transcriptions using SuperScript III Reverse Transcriptase (Thermo Fisher Scientific., lnc.), qPCR mixtures were prepared using QuantiTect SYBR Green RT-PCR Kit (QIAGEN, Germany). Primers of lncRNA CASC2 and endogous control β-actin were designed and synthesized by Sangon (Shanghai, China). Expression of lncRNA CASC2 was normalized to endogenous control β-actin using 2^−ΔΔCT^ method.

### ELISA

Plasma and synovial fluid levels of IL-17 were measured using Human IL-17 Quantikine ELISA Kit (D1700, R&D Systems). All steps were completed according to the instructions of the kit. Plasma levels of IL-17 were normalized to pg/ml.

### Cell line, cell culture, vectors and cell transfection

CHON-001 human chondrocyte cell line (ATCC, USA) was used. Dulbecco’s Modified Eagle’s Medium containing 0.1 mg/ml G-418 and 10% FBS was cell culture medium and cell culture conditions were 37°C and 5% CO_2_. Vectors expressing lncRNA CASC2 were designed and synthesized by Sangon (Shanghai, China). Cell transfections were performed using lipofectamine 2000 reagent to transfect 10 nM either lncRNA CASC2 vector or empty vector (negative control) in to 10^5^ cells. Cells with no transfections were control cells.

### Cell proliferation assay

RT-qPCR was performed at 24 h after transfection to check the overexpression rate of lncRNA CASC2. Cell proliferation was checked using Cell Counting Kit-8 (CCK-8, Dojindo Molecular Technologies, Inc.) only in cases of overexpression rate when lncRNA CASC2 was above 200%. In Brief, CHON-001 cell suspensions were prepared and cell density was adjusted to 4×10^4^ cells/ml. Cell suspensions were transferred to a 96-well plate with 4×10^3^ cells in each well. The plate was kept in an incubator (37°C, 5% CO_2_), and CCK-8 solution (10 μl) was added 24, 48, 72 and 96 h after the beginning of incubation. After that cells were cultivated from additional 4 h and 200 μl of DMSO was added. OD values at 450 nm were measured.

### Cell apoptosis assay

RT-qPCR was performed at 24 h after transfection to check the overexpression rate of lncRNA CASC2. Cell apoptosis was checked by cell apoptosis assay only in cases of overexpression rate of lncRNA CASC2 was above 200%. In Brief, CHON-001 cell suspensions were prepared and cell density was adjusted to 4×10^4^ cells/ml. Cell suspensions were transferred to a six-well plate with 10 ml in each well. Cells were cultivated in an incubator (37°C, 5% CO_2_) for 48 h. After 0.25% trypsin digestion, cells were subjected to Annexin V-FITC and propidium iodide (PI) (Dojindo, Japan) and flow cytometry was performed to detect apoptotic cells.

### Western blot

CHON-001 cells (10^5^) were mixed with 1 ml RIPA solution (Beyotime, Jiangsu, China) from 10^6^ CHON-001. After that, protein samples were denatured and subjected to 10% SDS-PAGE gel electrophoresis. After gel transfer to PVDF membrane, membranes were blocked in non-fat milk (5%) for 2 h at 22°C. After that, membranes were incubated with IL-17 (1: 1600, ab79056, Abcam) and GAPDH (1:1200, ab9485, Abcam) primary rabbit polyclonal primary antibodies, followed by incubation with IgG-HRP (goat anti-rabbit, 1:1000, MBS435036, MyBioSource) secondary antibody. Signals were detected using ECL reagent (Sigma–Aldrich, USA). Data normalization was performed using Image J v1.46 software.

### Statistical analysis

All *in vivo* and *in vitro* experiments included three biological replicates. Data analyses were performed using Graphpad Prism 6 software. Pearson’s correlation coefficient was used to analyze the correlations between expression levels of lncRNA CASC2 and IL-17 in plasma of both patients and healthy controls. Unpaired *t* test was used to analyze differences between two groups. One-way ANOVA and Tukey test were used to analyze differences among three groups. *P*<0.05 was statistically significant.

## Results

### LncRNA CASC2 and IL-17 were both up-regulated in osteoarthritis patients

**Figure 1:**
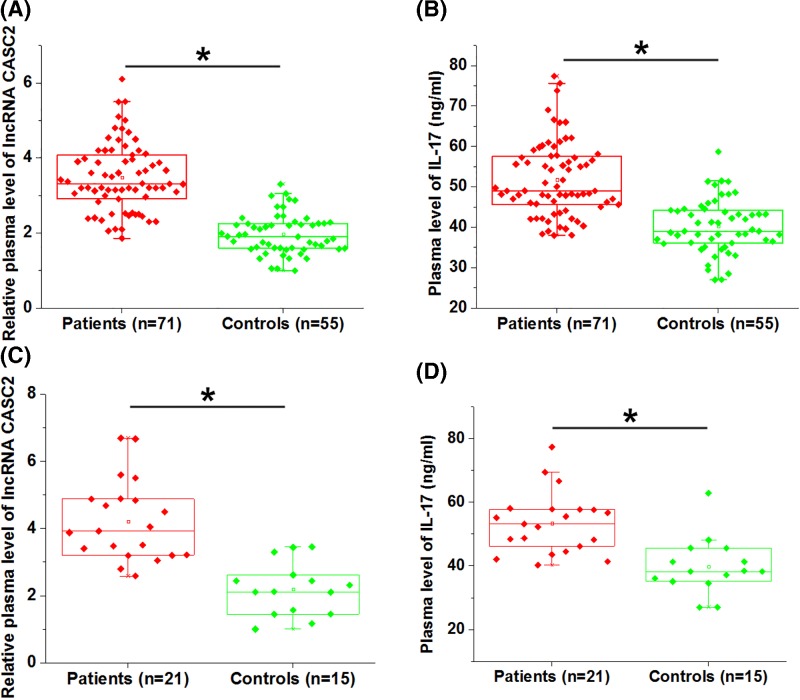
LncRNA CASC2 and IL-17 were both up-regulated in plasma of osteoarthritis patients RT-qPCR and ELISA results showed that, compared with healthy controls, both lncRNA CASC2 (**A**) and IL-17 (**B**) were up-regulated in plasma of osteoarthritis patients. In addition, lncRNA CASC2 (**C**) and IL-17 (**D**) were both up-regulated in synovial fluid of osteoarthritis patients comparing to healthy control. Each solid square represents the mean value of three biological replicates (*P*<0.05).

Plasma levels of lncRNA CASC2 and IL-17 in osteoarthritis patients (*n* = 71) and healthy controls (*n* = 55) were measured. Comparing to control group, lncRNA CASC2 (Figure 1A) and IL-17 (Figure 1B) were both up-regulated in plasma of osteoarthritis patients (*P*<0.05). Besides that, lncRNA CASC2 and IL-17 in synovial fluid of 21 osteoarthritis patients and 15 healthy controls were also detected. Similarly, lncRNA CASC2 (Figure 1C) and IL-17 (Figure 1D) were both up-regulated in synovial fluid of osteoarthritis patients comparing to healthy controls (*P*<0.05).

### LncRNA CASC2 and IL-17 were correlated

**Figure 2:**
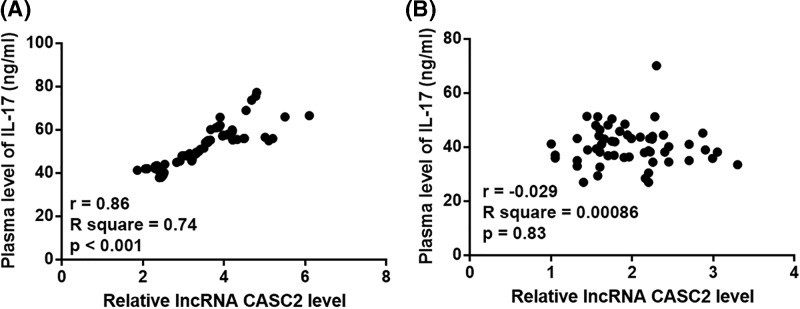
Plasma levels of lncRNA CASC2 and IL-17 were significantly and positive correlated only in osteoarthritis patients Pearson’s correlation coefficient showed that plasma levels of lncRNA CASC2 and IL-17 were significantly and positive correlated in osteoarthritis patients (**A**), but not in healthy controls (**B**).

Pearson’s correlation coefficient was used to analyze the correlations between expression levels of lncRNA CASC2 and IL-17 in plasma of both patients and healthy controls. Results showed that plasma levels of lncRNA CASC2 and IL-17 were significantly and positive correlated in osteoarthritis patients (Figure 2A). However, lncRNA CASC2 and IL-17 were significantly correlated in healthy controls (Figure 2B).

### Overexpression of lncRNA CASC2 led to up-regulated IL-17 expression in chondrocytes

**Figure 3:**
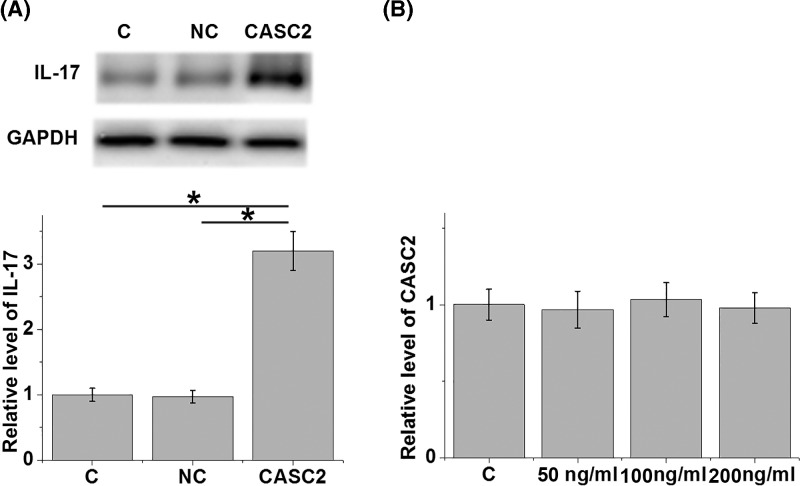
Overexpression of lncRNA CASC2 led to up-regulated IL-17 expression in chondrocytes Western blot results showed that overexpression of lncRNA CASC2 (CASC2) led to up-regulated IL-17 expression in chondrocyte (**A**) (*, *P*<0.05). In contrast, RT-qPCR results showed that treatment with exogenous IL-17 (SRP3080, Sigma–Aldrich) at doses of 10, 20 and 30 ng/ml for 24 h failed to significantly affect lncRNA CASC2 expression (**B**). Error bars indicate standard deviation.

After lncRNA CASC2 transfection, comparing to two controls (control, C, and negative control, NC), overexpression of lncRNA CASC2 (CASC2) led to up-regulated IL-17 expression in chondrocyte (Figure 3A, *P*<0.05). Moreover, IL-17 (SRP3080, Sigma–Aldrich) treatment at doses of 50, 100 and 200 ng/ml for 24 h failed to significantly affect lncRNA CASC2 expression (Figure 3B).

### LncRNA CASC2 regulated proliferation and apoptosis of chondrocytes

**Figure 4:**
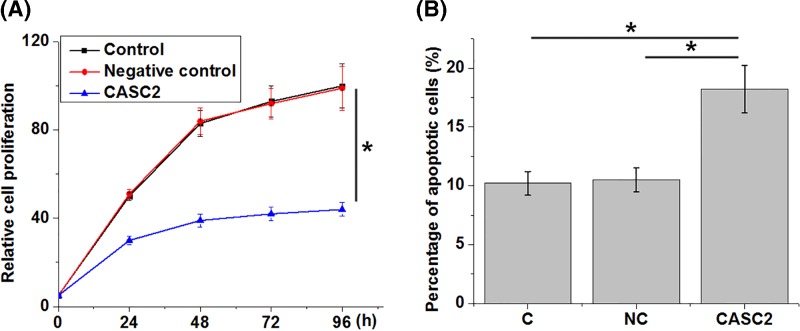
Overexpression of lncRNA CASC2 inhibited the proliferation and promoted the apoptosis of chondrocyte Cell proliferation assay and apoptosis assay revealed that, compared with the control (C) and negative control (NC) groups, overexpression of lncRNA CASC2 (CASC2) led to significantly inhibited proliferation (**A**) and promoted (**B**) apoptosis of chondrocytes. Error bars indicate standard deviation (*P<0.05).

To further explore the roles of lncRNA CASC2 in osteoarthritis, proliferation and apoptosis of chondrocytes were detected by cell proliferation assay and apoptosis assay after the overexpression of lncRNA CASC2. Compared with the control (C) and negative control (NC) groups, overexpression of lncRNA CASC2 (CASC2) led to significantly inhibited proliferation (Figure 4A) and promoted (Figure 4B) apoptosis of chondrocytes (*P*<0.05).

## Discussion

LncRNA CASC2 has been well studied in the development of different types of human cancers [15,16]. However, the functionality of lncRNA CASC2 in other diseases is still hardly known. The present study first reported the involvement of lncRNA CASC2 in osteoarthritis and further confirmed that the actions of lncRNA CASC2 in this disease are likely achieved by interacting with IL-17.

LncRNA CASC2 is down-regulated during the development of cancers [15,16]. The down-regulation of lncRNA CASC2 participates in the regulation of multiple cellular behaviors of cancer cells, such as proliferation and apoptosis [17,18]. In the present study we first reported that lncRNA CASC2 was up-regulated in osteoarthritis patients than in healthy controls. In addition, our *in vitro* cell experiment also proved that lncRNA CASC2 overexpression led to inhibited proliferation and promoted apoptosis of chondrocytes. It is known that the apoptosis of chondrocytes significantly contributed to the development of osteoarthritis [19]. Therefore, the overexpression of lncRNA CASC2 may promote the development of osteoarthritis by promoting the apoptosis of chondrocytes.

Osteoarthritis is an inflammatory disease. IL-17 as pro-inflammatory factor promotes the development of osteoarthritis by inducing inflammatory responses [20]. In effect, inhibition of IL-17 assists the treatment of osteoarthritis [21]. Consistently, this study also observed significantly up-regulated IL-17 in osteoarthritis than in healthy people. In has been reported that the expression of IL-17 can be regulated in lncRNA [22]. In the present study we proved that lncRNA CASC2 was likely an upstream activator of IL-17. However, the up-regulation of IL-17 by lncRNA CASC2 is likely mediated by pathological factors due to the fact that expression levels of IL-17 and lncRNA CASC2 were not significantly correlated in healthy controls. Therefore, overexpression of lncRNA CASC2 may also participate in osteoarthritis by up-regulating pro-inflammatory factor IL-17.

In conclusion, lncRNA CASC2 and IL-17 are up-regulated in osteoarthritis. LncRNA CASC2 may also participate in osteoarthritis by inducing cell apoptosis and up-regulating pro-inflammatory factor IL-17.

## Informed consent

All patients provided written informed consent.

## Abbreviations

CCK-8: Cell Counting Kit-8
lncRNA: long non-coding RNA

## References

[1] Loeser R.F., Goldring S.R. and Scanzello C.R. (2012) Osteoarthritis: a disease of the joint as an organ. Arthritis Rheumatol. 64, 1697–1707 10.1002/art.34453 PMC3366018 22392533

[2] McInnes I.B. and Schett G. (2011) The pathogenesis of rheumatoid arthritis. N. Engl. J. Med. 365, 2205–2219 10.1056/NEJMra1004965 22150039

[3] Pereira D., Peleteiro B., Araujo J., Branco J., Santos R.A. and Ramos E. (2011) The effect of osteoarthritis definition on prevalence and incidence estimates: a systematic review. Osteoarthritis Cartilage 19, 1270–1285 10.1016/j.joca.2011.08.009 21907813

[4] Bijlsma J.W., Berenbaum F. and Lafeber F.P. (2011) Osteoarthritis: an update with relevance for clinical practice. Lancet 377, 2115–2126 10.1016/S0140-6736(11)60243-2 21684382

[5] Lane N.E., Schnitzer T.J., Birbara C.A., Mokhtarani M., Shelton D.L., Smith M.D. (2010) Tanezumab for the treatment of pain from osteoarthritis of the knee. N. Engl. J. Med. 363, 1521–1531 10.1056/NEJMoa0901510 20942668 PMC6896791

[6] Saltzman C.L., Kadoko R.G. and Suh J.S. (2010) Treatment of isolated ankle osteoarthritis with arthrodesis or the total ankle replacement: a comparison of early outcomes. Clin. Orthop. Surg. 2, 1–7 10.4055/cios.2010.2.1.1 20190994 PMC2824089

[7] Man G.S. and Mologhianu G. (2014) Osteoarthritis pathogenesis - a complex process that involves the entire joint. J. Med. Life 7, 37–41 24653755 PMC3956093

[8] Berenbaum F. (2013) Osteoarthritis as an inflammatory disease (osteoarthritis is not osteoarthrosis!). Osteoarthritis Cartilage 21, 16–21 10.1016/j.joca.2012.11.012 23194896

[9] Goldring M.B. and Otero M. (2011) Inflammation in osteoarthritis. Curr. Opin. Rheumatol. 23, 471–478 10.1097/BOR.0b013e328349c2b1 21788902 PMC3937875

[10] Liu Y., Peng H., Meng Z. and Wei M. (2015) Correlation of IL-17 level in synovia and severity of knee osteoarthritis. Med. Sci. Monit. 21, 1732–1736 10.12659/MSM.893771 26076201 PMC4480114

[11] Chabaud M., Garnero P., Dayer J.M., Guerne P.A., Fossiez F. and Miossec P. (2000) Contribution of interleukin 17 to synovium matrix destruction in rheumatoid arthritis. Cytokine 12, 1092–1099 10.1006/cyto.2000.0681 10880256

[12] Fatica A. and Bozzoni I. (2014) Long non-coding RNAs: new players in cell differentiation and development. Nat. Rev. Genet. 15, 7–21 10.1038/nrg3606 24296535

[13] Shi X., Sun M., Liu H., Yao Y. and Song Y. (2013) Long non-coding RNAs: a new frontier in the study of human diseases. Cancer Lett. 339, 159–166 10.1016/j.canlet.2013.06.013 23791884

[14] Song J., Ahn C., Chun C.H. and Jin E.J. (2014) A long non-coding RNA, GAS5, plays a critical role in the regulation of miR-21 during osteoarthritis. J. Orthop. Res. 32, 1628–1635 10.1002/jor.22718 25196583

[15] Pei Z., Du X., Song Y., Fan L., Li F., Gao Y. (2017) Down-regulation of lncRNA CASC2 promotes cell proliferation and metastasis of bladder cancer by activation of the Wnt/beta-catenin signaling pathway. Oncotarget 8, 18145–18153 10.18632/oncotarget.15210 28199978 PMC5392314

[16] Liao Y., Shen L., Zhao H., Liu Q., Fu J., Guo Y. (2017) LncRNA CASC2 interacts with miR-181a to modulate glioma growth and resistance to TMZ through PTEN pathway. J. Cell. Biochem. 118, 1889–1899 10.1002/jcb.25910 28121023

[17] Fan J.C., Zeng F., Le Y.G. and Xin L. (2018) LncRNA CASC2 inhibited the viability and induced the apoptosis of hepatocellular carcinoma cells through regulating miR-24-3p. J. Cell. Biochem. 119, 6391–6397 10.1002/jcb.26479 29091305

[18] Li P., Xue W.J., Feng Y. and Mao Q.S. (2016) Long non-coding RNA CASC2 suppresses the proliferation of gastric cancer cells by regulating the MAPK signaling pathway. Am. J. Transl. Res. 8, 3522–3529 27648142 PMC5009404

[19] Blanco F.J., Guitian R., Vazquez-Martul E., de Toro F.J. and Galdo F. (1998) Osteoarthritis chondrocytes die by apoptosis. A possible pathway for osteoarthritis pathology. Arthritis Rheum. 41, 284–289 10.1002/1529-0131(199802)41:2<284::AID-ART12>3.0.CO;2-T 9485086

[20] Holzer N., Salvo D., Marijnissen A.C., Vincken K.L., Ahmad A.C., Serra E. (2015) Radiographic evaluation of posttraumatic osteoarthritis of the ankle: the Kellgren-Lawrence scale is reliable and correlates with clinical symptoms. Osteoarthritis Cartilage 23, 363–369 10.1016/j.joca.2014.11.010 25463444

[21] Mimpen J.Y., Carr A.J. and Dakin S.G. (2018) Inhibition of interleukin-17-induced effects in osteoarthritis-an in vitro study. Osteoarthritis Cartilage 26, S118 10.1016/j.joca.2018.02.258

[22] Huang X.D., Dai J.G., Lin K.T., Liu M., Ruan H.T., Zhang H. (2018) Regulation of IL-17 by lncRNA of IRF-2 in the pearl oyster. Fish Shellfish Immunol. 81, 108–112 10.1016/j.fsi.2018.07.020 30017925

